# Medical education in china: progress in the past 70 years and a vision for the future

**DOI:** 10.1186/s12909-021-02875-6

**Published:** 2021-08-28

**Authors:** Weimin Wang

**Affiliations:** 1 Health Science Center, Peking University, No.38 Xueyuan Road, Haidian District, Beijing, P.R. China; 2 Institute of Medical Education & National Center for Health Professions Education Development, Peking University, Beijing, P.R. China; 3 Working Committee for the Accreditation of Medical Education, Ministry of Education, Beijing, P.R. China

**Keywords:** Medical education, Health professional, Reform, China

## Abstract

Medical education in China has undergone significant reforms in contemporary times. As the world’s largest medical education system, it is important to understand the status of China’s medical education in our interdependent world. This paper highlights the current landscape of medical education in China, particularly the progress that have been made in recent years. It also examines the current topics and challenges facing China’s medical educators today, and proposed recommendations for improving medical education in China. The medical education in China will produce better qualified health professionals to meet the health needs of Chinese population according to the new requirements of the “Healthy China 2030” blueprint.

## Background

As the most populous country and the second largest economy in the world, China has built the world’s largest medical education system since the People’s Republic of China was founded in 1949. With the deepening of China’s health-care system reform, China is striving to modernise and standardise its medical education, and has experienced several epoch-making stages. Starting in 1998, stand-alone medical institutions were merged into comprehensive universities [1]. The accreditation of clinical medical education was established in 2008 [2]. The standardized residency training (SRT) was launched as a national strategy in 2013 [3]. In 2020, the Working Committee for the Accreditation of Medical Education (WCAME) of China was recognized as the accreditation body by the World Federation for Medical Education (WFME) [2]. With the release of the “Healthy China 2030” blueprint, China elevates the maintenance of people’s health to the height of national strategy, and China’s medical education reform has entered a new era [4]. The COVID-19 epidemic has also placed new requirements for health professional education. In this commentary, I summarize the progress of medical education in China, focus on the system, scale, structure and quality of health professional education, discuss the challenges and countermeasures of medical education in the above aspects.

## Overview of medical education in China

### The scale and structure of medical professional education

China has the world’s largest medical education system. In 2018, China had 420 undergraduate institutions with medical education (excluding military institutions), matriculating 286,219 medical undergraduates, 81,128 masters and 14,044 doctors in 2018–2019.

**Fig. 1:**
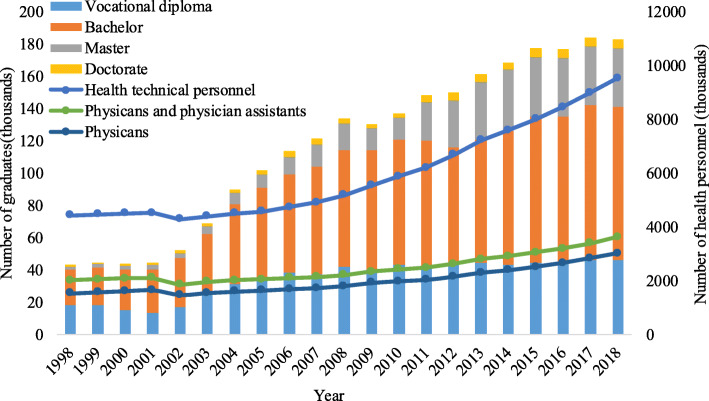
China’s health technical professionals and clinical medical graduates by education level, 1998–2018

The number of higher clinical medicine graduates rose dramatically from 51,800 in 2002, to 182,900 in 2018, with an average annual growth rate of 8.2 % between 2002 and 2018, of which undergraduate students increased from 30,500 to 94,600, with an average annual growth rate of 7.3 % (Fig. 1). Medical education provides strong support for health care demands of the Chinese people. At present, the number of health personnel per 1,000 population in China is above the level of moderately developed countries. By the end of 2019, China had 12.9 million healthcare workers, with 2.8 doctors and 3.2 nurses per 1,000 population. There were 6.4 health workers serving in public health institutions and 2.6 general practitioners per 10,000 population [5].

The educational background of Chinese medical graduates has been continuously improved. Take clinical medicine as an example, from 2015 to 2018, graduates with bachelor’s degrees or above accounted for 75 % of the higher clinical medical graduates. Higher medical education, which includes the medical education in junior college, undergraduate and graduate education, has made a key contribution to the improvement of the educational degrees of China’s health personnel (Fig. 1). The structure of health workers has also been optimized. China had more doctors than nurses before 2014, but this situation has been improved. At the end of 2019, the national ratio of doctors to nurses was 1:1.15 [5].

## Clinical medical education system

China has gradually established a clinical medical education system with Chinese characteristics, which includes three stages of medical school, graduate medical education and continuing education, with “5 + 3” model as the main body, 8-year program as the exploration and “3 + 2” model as the supplement [6].

In a broad sense, there are three types of “5 + 3” model. The first encompasses 5 years of undergraduate medical education (leading to a bachelor degree) and 3 years of standardized residency training (SRT). The second is finishing 5 years of undergraduate education first, then taking the postgraduate entrance examination and completing 3 years of professional master degree (master of medicine, MM) programme (including SRT). The third is that the 5 years undergraduate study is followed by 3 years of MM programme, without taking postgraduate examination. The three types will gradually realize the connection between professional graduate education and professional qualification by integrating the SRT and the equivalent professional master programme. The “3 + 2” model includes 3 years of higher vocational clinical medical education and 2 years of assistant general practitioner training, aiming at cultivating clinical assistant physicians.

In addition, there is an 8-year program starting from senior high school for producing high-level innovative medical professionals. The 8-year program originated from Peking Union Medical College and expanded from Peking University Health Science Centre to other high-level universities in 2001. The program adopts 8-year integrated mode in which the graduates will be granted bachelor and doctor degree. In September 2018, Peking Union Medical College began to pilot the 4 (non-medical education) + 4 (clinical medicine) education program starting from the undergraduate level [7], which is similar to the US Medical Doctor (MD) program.

As of September 2018, among the 192 universities with clinical medicine majors, 151 universities had 5-year programmes, with a total annual enrolment of about 87,000. There were 27 universities offering “5 + 3” integrated programmes, with a total enrolment of about 4,900 students annually. There were 14 universities offering 8-year programmes, and the total annual enrolment was about 1,100 students (Table 1).

**Table 1:** College-level medical education programmes and matriculates in China, 2018

Programme	Degrees	Number of medical schools	Matriculants/year(1000 s)	Time of clinical rotations	Professional role
5-year Programme	Bachelor	151^a^	87±	3 years	Specialist/General
5 + 3 integration programme	Master	27^b^	4.9±	3 years	Specialist
8-year programme	Doctor	14^b^	1.1±	1 ~ 2 years	Specialist
Total		192	93±		

^a^Schools that only provide 5-year programmes; ^b^Schools that also provide 5-year programmes

## Establishment and implementation of medical education accreditation

Accreditation of medical education is an internationally accepted quality assurance system and an important foundation for achieving international recognition and the cross-border flow of doctors. In 2006, China started the pilot accreditation based on the *Global Standards for Basic Medical Education* of WFME. In 2008, the “*Accreditation Standards for Basic Medical Education in China (Trial)*” were issued and the Working Committee for the Accreditation of Medical Education (WCAME) of Ministry of Education (MoE) was established. In 2017, the 2016 edition of the standards was released [2]. As of December 2019, the first round of accreditation for clinical medicine in 105 medical schools had been completed, accounting for 75.5 % of the institutions, which supposed to be accredited. In June 2020, WCAME of China was recognized as the accreditation body by WFME, which marked that the professional accreditation of clinical medicine with Chinese characteristics is equivalent to the international accreditation [2].

## Reform of training modes of medical education in China

China has been continuously exploring the reform of training mode in medical education through optimizing educational objectives, integrating teaching contents, enriching teaching modes, improving education system, and innovating evaluation and assessment system [8]. Great importance has been attached to the cultivation of professionalism and innovation. Currently, China is actively promoting the competence-based medical education with the aim of improving the quality of medical students training[9].

According to a survey conducted by the National Center for Health Professionals Education Development at Peking University, during the 2018–2019 academic year, 14.6 % of institutions implemented problem-based learning, and 15.7 % of institutions adopted disciplinarily integrated curricula.

Taking Peking University Health Science Center (PKUHSC) as an example, the “new pathway” medical education reform was implemented in 2008, which realized the shift of curricula from subject-centered to the organ-system-centered, and learning in small groups was implemented. In 2019, the “New Era” medical education reform was launched in PKUHSC, aiming to realize the integration of basic and clinical medical courses, and the development of students’ independent learning capability was paid more attention.

## Financial support for Chinese medical institutions

Since the 21st century, the investment in medical education has increased significantly. From 2008 to 2012, the annual grant per student increased from 12,000 CNY ($1,767.6) to 27,000 CNY($3,977.1) for 23 institutions supervised by MoE, reaching the highest standard per student among all majors [10]. Further improvements are expected in the future. Under the guidance of the central government, many provinces have raised their own average funding per student for medical majors. The provincial government’s investment is the main channel for the funds sources of local medical schools .

## Critical issues and challenges for medical education in China

The undergraduate and graduate medical education are supervised by national and provincial education departments, while the standardized resident training and continuing medical education are supervised by health department at national and provincial level. Although the Chinese government is actively promoting the cooperation between health and education sectors, which is commonly called “Medicine-Education Cooperation”, there is still a lack of effective coordination mechanism between the education sector (supplier) and the health sector (demander) [11]. In addition, at the level of institutions, there is a contradiction between the emphasis on research and the underestimation of medical education in the construction of world top universities and disciplines [12].

As the largest developing country, China’s economic development is unbalanced, and the quality of medical education in the underdeveloped regions is still far below the desired standard of undergraduate medical education in China. In addition, China’s health professional education system is complex and consists of degree programmes lasting from 3 to 8 years. The coexistence of 5-year and 8-year programmes with programmes of shorter duration can help serve the specialist demands for health care without sacrificing the needs of population for basic and primary health care [13]. However, the inconsistency of the various training tracks has led to uneven quality of physicians, and has negative effects on the forming of high quality and reliable medical system. It is also unconducive to the equalization and fairness of the health system in China.

The expansion of school enrolment leads to the decline of the quality of health professional education, for the faculty numbers and other teaching resources have failed with numeric expansion of students [1]. In 2018, 186 medical institutes in China enrolled about 93,000 students studying clinical medicine, with an average enrolment of about 500 per school. Under the condition of certain educational resources per institute, the excessive enrolment scale will inevitably affect the quality of medical education [14].

In addition to higher medical universities, China also provides health professional education in junior medical colleges, in which 3-year education after senior high school can lead to a vocational diploma. These vocational medical colleges aims to train nurse, allied health professionals while medical doctors usually trained in more competitive universities. As for clinical medicine in 2018, nearly 15 % of graduates have the vocational diploma, which are lower than a bachelor degree. The quantity and quality of professionals in public health and preventive medicine are not enough to meet the needs of “Health China 2030” blueprint [15].

In the educational process of medical education, medical schools are slow in responding to external demands and the training modes are inflexible. Medical education managers and teachers lag behind in updating their education concepts. Student-centered and competence-based medical education are not widely implemented. The curriculum still focuses narrowly on biomedicine, medical technology and clinical practice. The pedagogic methods are mostly teacher-controlled didactic lecturing, which is ineffective when compared to modern teaching that incorporates active learning [1].

Due to the declining attraction to medicine as a career and professional identity, a growing number of outstanding young people are losing their enthusiasm to study medicine, and there is a declining interest in pursuing career opportunities in medical industry among medial graduates [16]. The reform process of personnel compensation system lacks breakthrough innovation. The incentive mechanism still needs to be improved. The relatively low income, difficult career promotion, heavy workload and low social status in public health services leads to serious brain drain of the key technical personnel in this field [15]. In addition, the research-oriented physician evaluation system is not suited to occupational characteristics.

## Recommendations for improving medical education in China

As a national strategy, the “Healthy China 2030” blueprint sets a goal of enabling everyone to be involved in health, share health, and be responsible for health. Realising the goal requires a number of qualified health personnel. Health professional education should be attached greater importance in this long-term national strategic plan. The Medicine-Education Cooperation mechanism at the level of the State Council, China’s cabinet, should be established, to lead the coordinated development of medical education and health services, and strengthen the management of the whole process of medical education [11]. Great emphasis should be placed on rebalancing the roles of comprehensive universities and its medical schools, granting more autonomy and authority to medical institutions to conduct medical education [12].

Based on the situation of China, promotion of the reform of educational programmes can be divided into 3 stages. In the first stage, the level of standardized residency training (SRT) institutions should be improved, so as to realize a homogenised and high-quality education. The second stage implements standardised residency training and clinical professional degree for all resident trainees to realize the “three certificates in one”, which means the trainees will receive master degree certificate, physician qualification certificate and SRT certificate after qualification from the residency. In the third stage, the master degree is cancelled and the Medical Doctor (MD) is awarded instead. The 8-year MD programme could be adjusted to 10 years, aiming at producing “physician scientists” with MD and PhD degree, which is similar to US MD-PhD joint programmes [11].

A supply-demand balance mechanism for health professional education should be established, which could reasonably determine the scale, structure and distribution of medical professional education [15]. The number of medical institutions should be increased appropriately and the enrolment scale of each school should be reduced. Improve the level of clinical medicine education, and gradually reduce the enrolment of junior medical colleges. At the same time, expand the enrolment scale of 5 + 3 integrated programmes and make it the main form of medical education.

The Lancet Commission on Education of Health Professionals for the 21st century summarise the emphasis of the third generation of educational reforms, which include patient and population centeredness, competency-based curriculum, interprofessional and team-based education [17]. All these experiences and practice should be used for reference and adopted in China’s medical education. For example, the “discipline-centered” curricula can be transformed to “organ-system-centered” integrated curricula. Medical humanities and professionalism education should be provided during education to promote the integration of humanities education and professional education. More emphasis should be placed on the cultivation of innovative thinking, critical thinking and collaboration, and improving the competence of medical graduates.

On the basis of accreditation of clinical medical education, accreditation should be horizontally extended to all health professions, such as nursing, public health and preventive medicine, and stomatology, and vertically extended to postgraduate medical education and continuing medical education, so as to accelerate the formation of a more complete medical accreditation system with Chinese characteristics. As for financing, the annual appropriation per student for medical education should be no less than 5 times that for other majors.

## Conclusions

With the rising needs from the healthcare system, China has launched ambitious reforms of its medical education to cultivate physicians with the professional, clinical competencies and humanistic spirit, which are essential for the quality of clinical services demanded by Chinese people with the improvement of living standards. However, there are still many challenges that China will have to overcome to produce qualified health professionals.

Medical education in China should continue to deepen the reform, in accordance with the new requirements of the “Healthy China 2030” blueprint. The medical education management system and mechanism should be further perfected for smooth operation based on the improved Medicine-Education Cooperation. The scale, structure and layout of medical professionals training should be optimized according to the needs of the healthcare system. The personnel training mode should be transformed to improve the competence of medical students by following the principles of medical education and the accreditation standards for clinical medical education. Medical education in China will produce better qualified health professionals to meet the health needs of Chinese population and provide powerful support for building a prosperous society in all respects.

## Data Availability

Not applicable.

## Abbreviations

MoE: Ministry of Education
PKUHSC: Peking University Health Science Center
SRT: Standardized residency training
WCAME: Working Committee for the Accreditation of Medical Education
WFME: World Federation for Medical Education
